# Supporting Weight Management during COVID-19 (SWiM-C): twelve-month follow-up of a randomised controlled trial of a web-based, ACT-based, guided self-help intervention

**DOI:** 10.1038/s41366-022-01232-x

**Published:** 2022-11-11

**Authors:** Julia Mueller, Rebecca Richards, Rebecca A. Jones, Fiona Whittle, Jennifer Woolston, Marie Stubbings, Stephen J. Sharp, Simon J. Griffin, Jennifer Bostock, Carly A. Hughes, Andrew J. Hill, Clare E. Boothby, Amy L. Ahern

**Affiliations:** 1 MRC Epidemiology Unit, University of Cambridge, Cambridge, UK; 2 Primary Care Unit, Department of Public Health and Primary Care, University of Cambridge, Cambridge, UK; 3 Patient and Public Involvement representative, Cambridge, UK; 4 Fakenham Medical Practice, Fakenham, UK; 5 Medical School, University of East Anglia, Norwich, UK; 6 Division of Psychological and Social Medicine, School of Medicine, University of Leeds, Leeds, UK

**Keywords:** Weight management, Lifestyle modification

## Abstract

**Objectives:**

We developed a guided self-help intervention (Supporting Weight Management during COVID-19, “SWiM-C”) to support adults with overweight or obesity in their weight management during the COVID-19 pandemic. This parallel, two-group trial (ISRCTN12107048) evaluated the effect of SWiM-C on weight and determinants of weight management over twelve months.

**Methods:**

Participants (≥18 years, body-mass-index ≥25 kg/m^2^) were randomised to the SWiM-C intervention or to a standard advice group (unblinded). Participants completed online questionnaires at baseline, four months, and twelve months. The primary outcome was change in self-reported weight from baseline to twelve months; secondary outcomes were eating behaviour (uncontrolled eating, emotional eating, cognitive restraint of food intake), experiential avoidance, depression, anxiety, stress, wellbeing and physical activity.

**Interventions:**

SWiM-C is based on acceptance and commitment therapy (ACT). Participants had access to an online web platform with 12 weekly modules and email and telephone contact with a trained, non-specialist coach. Standard advice was a leaflet on managing weight and mood during the COVID-19 pandemic.

**Results:**

388 participants were randomised (SWiM-C: *n* = 192, standard advice: *n* = 196). The baseline-adjusted difference in weight change between SWiM-C (*n* = 119) and standard advice (*n* = 147) was −0.81 kg (95% CI: −2.24 to 0.61 kg). SWiM-C participants reported a reduction in experiential avoidance (−2.45 [scale:10–70], 95% CI: −4.75 to −0.15), uncontrolled eating (–5.52 [scale: 0–100], 95% CI: –9.67 to –1.37), and emotional eating (–4.49 [scale: 0–100], 95% CI: –7.57 to –1.42) and an increase in physical activity (8.96 [MET-min/week], 95% CI: 0.29 to 17.62) compared to standard advice participants. We found no evidence of an effect on remaining outcomes. No adverse events/side effects were reported.

**Conclusions:**

Whilst we were unable to conclude that the intervention had an effect on weight, SWiM-C improved eating behaviours, experiential avoidance and physical activity. Further refinement of the intervention is necessary to ensure meaningful effects on weight prior to implementation in practice.

**Trial registration number:**

ISRCTN 12107048

## Introduction

Behavioural weight management interventions can lead to reductions in weight and improvements in health outcomes, but effects are often small and short-term [1]. A recent systematic review and network meta-analysis found that interventions that incorporate strategies based on acceptance and commitment therapy (ACT) may have better long-term weight outcomes than standard behavioural treatment [2]. ACT posits that individuals engage in unhelpful behaviour (e.g., overeating) to avoid uncomfortable internal experiences (e.g., negative feelings, food cravings). It aims to help people deal with such experiences more flexibly using mindfulness and acceptance skills (i.e., through increased psychological flexibility) and commit to behaviour consistent with their values.

Inexpensive, scalable weight management interventions are urgently needed. However, ACT-based interventions are usually delivered in-person by clinical psychologists who are a scarce and expensive commodity, thereby limiting scalability. Evidence on remotely delivered ACT-based weight management interventions is currently scarce; a recent systematic review identified only three studies [2], and these were not designed to detect effects on weight [3–5]. Studies published since the review are restricted to feasibility trials with small samples or pre-post studies that do not allow causal inferences [6–8].

The need for inexpensive, scalable interventions for behavioural weight management became particularly urgent during the COVID-19 pandemic, when social distancing and isolation measures meant that adults with overweight and obesity had reduced access to weight management interventions and services. Evidence from the early stages of the pandemic also suggests that adults with overweight and obesity were vulnerable to weight gain and changes in eating behaviour (e.g., increased emotional eating) [9–11]. Weight gain is associated with increased risk factors for diabetes, cardiovascular disease and related disorders [12] and studies show that discrete periods of weight gain (such as those that occur during holidays) are not fully compensated and can lead to considerable weight gain over time [13]. Additionally, obesity is an independent risk factor for severe forms of COVID-19 [14].

In response to the need for remotely delivered support, we developed an ACT-based, guided self-help intervention (Supporting Weight Management during COVID-19, “SWiM-C”) to help adults with overweight and obesity manage their weight and eating behaviour, be more physically active, and protect their emotional wellbeing during the COVID-19 pandemic [15]. SWiM-C was a three-month programme delivered using remote technology to improve cost, scalability and reach of the intervention. It was, therefore, well suited for delivery during the pandemic but also for other situations involving reduced access to resources and/or low levels of mobility. SWiM-C used strategies derived from ACT to increase psychological flexibility and reduce experiential avoidance, i.e. to increase individuals’ willingness to experience uncomfortable feelings (e.g., food cravings) in order to pursue their values (e.g., being healthy). It is hypothesised that this facilitates improved behavioural responses (e.g., reductions in emotional and uncontrolled eating). SWiM-C sought to improve wellbeing and mental health and promote physical activity because we hypothesised that the COVID-19 pandemic would have a negative effect on these variables, thereby increasing risk for weight gain. Evidence has since confirmed that the pandemic and related restriction measures are likely to have affected mental health, wellbeing, physical activity and weight [10, 16, 17].

In a randomised controlled trial with adults with overweight or obesity, we found that at four months post-baseline, SWiM-C led to improvements in psychological determinants associated with improved weight management (increased psychological flexibility and cognitive restraint of food intake, reduced uncontrolled eating), and had a protective effect on wellbeing compared to a standard advice control group [15]. Results were inconclusive regarding effects on bodyweight [15].

Initially, we evaluated the SWiM-C intervention over 4 months [15] to ensure rapid evidence dissemination during the pandemic. To investigate longer-term effects of SWiM-C, we followed up participants from that trial at twelve months. The primary aim of the current study is to evaluate the effect of SWiM-C on weight change from baseline to twelve months compared to standard advice. The secondary aims were to evaluate the effect of SWiM-C over twelve months on determinants of weight management, including eating behaviour, physical activity, wellbeing, depression, anxiety, perceived stress, and experiential avoidance/psychological flexibility, compared to a standard advice control group. We also evaluated the effect of SWiM-C on change in weight from four months to twelve months to assess how outcomes changed from post-intervention to longer-term follow-up.

## Methods

### Trial design

This is a randomised, parallel, two-group trial. Participants were randomised to either the SWiM-C intervention or to a standard advice wait-list control group. Participants completed outcome assessments online at baseline, and at four months and twelve months post-baseline. A detailed description of trial methods is provided elsewhere [15]. Ethical approval was obtained from the Cambridge Psychology Research Ethics Committee (Application No: PRE.2020.049) on 24/04/2020. All participants gave written, informed consent. Clinical trial registration: ISRCTN 12107048.

### Eligibility criteria

Individuals were eligible to participate if they were adults with overweight or obesity (age ≥ 18 years; BMI ≥ 25 kg/m^2^), living in the UK, had a good understanding of written English, were willing to be randomised to either intervention or a standard advice group and to complete outcome assessments online, owned a set of bodyweight scales, and had not received bariatric surgery in the two years prior to the study.

### Recruitment

Participants were recruited using mailing lists and social media advertisements from local weight management services and obesity organisations (e.g. Obesity UK), and through a volunteer database from a previous study [18].

### Intervention

A detailed description of the intervention is provided elsewhere [15, 19]. Briefly, the intervention is an ACT-based, web-based, guided self-help intervention. It includes access to an online web platform with 12 weekly modules (SWiM sessions) consisting of psychoeducational content, reflective exercises, and behavioural experiments. After participants had completed session 4, they received a telephone call from their SWiM coach (a 20-minute call focusing on reviewing exercises, troubleshooting, and ensuring participants understood the content). The coach also sent a tailored email at week 10.

### Control

The wait-list control group received standard advice in the form of a leaflet from the European Association for the Study of Obesity (EASO) on diet, physical activity, and mood for people living with obesity during the COVID-19 pandemic [15]. They received access to the SWiM-C web platform after completing the 4 month outcome assessment but did not have telephone or email support from a coach.

### Randomisation

Participants were randomised to either the intervention (SWiM-C) or the standard advice group using a 1:1 allocation ratio with block randomisation (block size 6) stratified by BMI classification (25 to <30, 30 to <40, 40+ kg/m^2^) and sex (male, female). The randomisation sequence was computer-generated by the trial statistician and programmed into the database by the data manager. The sequence was unknown to research staff and participants.

### Procedure

Following informed consent via webform, participants completed baseline assessments using online questionnaires. Participants were then randomised to SWiM-C (participants received access to the SWiM-C intervention) or standard advice (participants were emailed the EASO leaflet). At four months and twelve months post-baseline, participants completed follow-up measures using online questionnaires. Participants were given honoraria for completing questionnaires (£10 for baseline and £20 respectively for the 4-month and 12-month follow-up). Honoraria for assessment completion were not dependent on intervention engagement.

To check the quality of data entry in the online surveys, we tabulated categorical variables and produced histograms of continuous variables to check whether distributions looked plausible. Additionally, webforms had built-in validation checks (e.g., for weight participants received a warning if the value was below 40 or above 200 but were able to continue if they confirmed). We assessed the data for biologically implausible weight change using the definition by Chen et al. [20].

### Sample size

The target sample for the trial was 360 participants to allow detection of a 1 kg difference in weight change between intervention and standard advice (assuming a standard deviation of 6 kg and a correlation between baseline and follow-up measures of 0.9) with 90% power and 95% confidence.

### Measures

#### Primary outcome

The primary outcome was change in self-reported weight from baseline to twelve-month follow-up (kg). Participants entered their weight into an online questionnaire. Participants were asked to weigh themselves on the day of questionnaire completion and were given detailed instructions on how to measure their weight at home (e.g. placing the scales on firm flooring, wearing light clothing).

#### Secondary outcomes

All outcomes were assessed using validated self-report questionnaires. Eating behaviour was measured using the Three-Factor Eating Questionnaire (TFEQ-R21) [21], which provides one score of 0-100 per subscale (cognitive restraint of food intake, uncontrolled eating, and emotional eating). Higher scores indicate higher restraint and more uncontrolled and emotional eating, respectively. Experiential avoidance/psychological flexibility was assessed using the Acceptance and Action Questionnaire Weight Related-Revised (AAQW-R) [22]. Scores on the AAQW-R range from 10 to 70, with higher scores indicating higher experiential avoidance and lower psychological flexibility. Volume of total physical activity in MET-min per week was measured using the International Physical Activity Questionnaire (IPAQ) [23]. We assessed three domains of mental health: depressive symptom severity using the Patient Health Questionnaire (PHQ-8 [24]; scores range from 0–24), anxiety symptom severity using the Generalized Anxiety Disorder 7-item scale (GAD-7 [25]; scores: 0–21), perceived stress using the Perceived Stress Scale (PSS-4 [26, 27]; scores: 0–16). Higher scores indicate higher symptom severity for depression, anxiety, and stress, respectively. Wellbeing/capability was assessed using the ICEpop CAPability measure for Adults (ICECAP-A [28], scores: 0–1, higher scores indicate higher wellbeing). Cronbach’s alpha values for all questionnaires are shown in Table S1 (supplement). Secondary outcomes also included change in self-reported weight from four months to twelve months.

At baseline, we also measured age (years), sex, ethnicity, educational qualifications, marital status, and height.

### Statistical analyses

Analyses were pre-specified in a statistical analysis plan (available at www.isrctn.com/ISRCTN12107048). To summarise baseline characteristics by randomised group, we present means and standard deviations (SDs) for continuous measures and the number and percentage of individuals within each category for categorical variables. Percentages were calculated using the number of non-missing values as the denominator.

The intervention effect on weight at twelve months (and 95% confidence interval [CI]) was estimated from a random intercepts linear regression model, using measures of change in weight from baseline to four months and twelve months as outcomes. The model included intervention group, assessment timepoint, the interaction term for group by assessment timepoint, the randomisation stratifiers (sex, BMI classification), and baseline value of weight as fixed effects, and random intercepts to allow for the repeated measures on each individual. Individuals were included in the analysis in the group to which they were randomised. Secondary outcomes were analysed using the same approach as described for the primary outcome.

Random intercept models use all available data and assume missing data are missing at random. To explore this assumption, we described baseline characteristics of participants with and without missing data on any outcome at twelve months. Because there were more missing values of weight in the intervention than in the standard advice group, we conducted a sensitivity analysis assuming data were missing not at random (MNAR) using pattern mixture models. Specifically, we imputed missing weight data using multiple imputation by chained equations (MICE). We then multiplied imputed values of weight by a varying factor (0% [MAR], or increasing or decreasing the values by 10%, 20%, 30%, [MNAR]) [29]. Subsequently we examined the estimates of the intervention effect under these 7 scenarios. If the influence under these scenarios is small, the analysis is considered to be robust against departures from the MAR assumption.

Participants in the wait-list standard advice group were given access to the SWiM-C web platform after the four-month outcome assessment. We therefore conducted per-protocol analyses excluding standard advice participants who engaged with the intervention. We compared intervention participants who engaged with the SWiM-C intervention with standard advice participants who did not engage with the SWiM-C intervention to assess whether the findings were influenced by the degree of engagement with the intervention. We defined non-engagement with the intervention as accessing less than the first four sessions (participants were only able to proceed through sessions sequentially), since ACT-based content was introduced from session 4. We performed two per-protocol analyses of the primary outcome with two different levels of engagement: 1) comparing intervention participants who completed at least 4 SWiM-C sessions with standard advice participants who accessed less than 4 SWiM-C sessions, and 2) comparing intervention participants who completed at least 8 SWiM-C sessions with standard advice participants who accessed less than 4 SWiM-C sessions.

To estimate baseline-adjusted differences between the study groups in change in weight from four months to twelve months, we used a linear regression model with change in weight from four months to twelve months as the outcome, and including baseline weight, four-month weight, the randomisation stratifiers (sex, BMI classification) and intervention group as covariates.

Data were analysed using R version 4.0.0 and R Studio version 1.0.153.

## Results

**Fig. 1:**
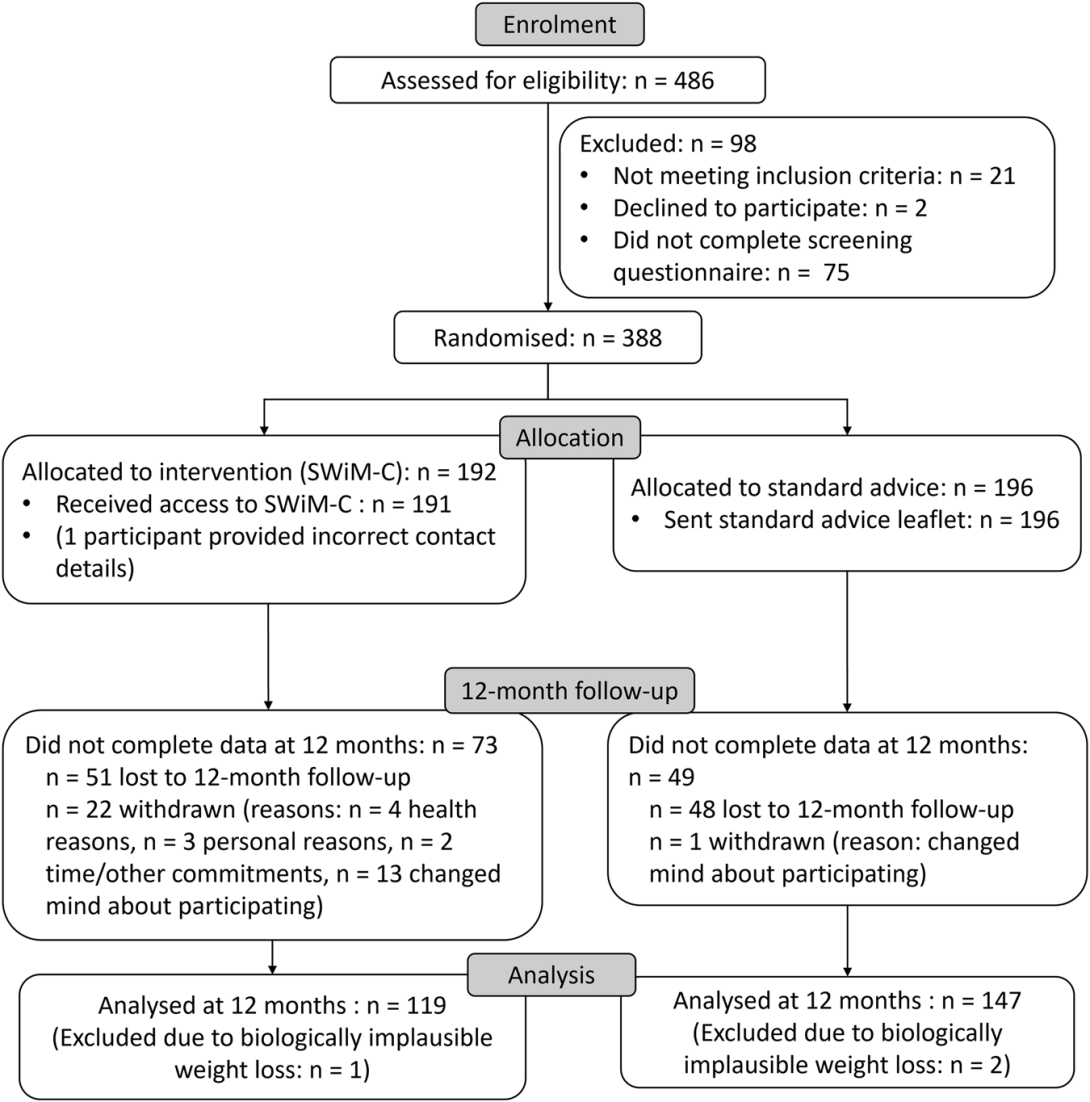
Consort flowchart. Consort flowchart showing the progress of participants through the phases of the trial. SWiM-C = Supporting Weight Management during COVID-19.

Overall, 486 participants were screened between 18th June 2020 and 7th September 2020, and 388 participants were randomised (intervention: *n* = 192, control: *n* = 196) (Fig. 1). Baseline characteristics of the sample are reported elsewhere [15]. The mean age was 50.7 (SD = 14.3) years and the mean BMI was 34.8 (7.7) kg/m^2^. Most participants identified as female (303/388, 78.1%), White (364/388, 93.8%), and had at least a university degree or equivalent (242/388, 62.4%). Participants in the intervention and standard advice groups did not differ substantially on baseline characteristics [15]. In terms of baseline mental health, mean scores at baseline for the PHQ-8, GAD-7 and PSS-4 were, respectively, 7.7 (SD = 5.5), 5.5 (SD = 5.0) and 6.5 (SD = 3.15). Means and SDs of outcomes by study group at baseline, four months, and twelve months are shown in Table S2 (supplement).

Data on weight were available for 266 (69.3%) participants at twelve months, after excluding three weight values due to biologically implausible weight loss [20]. Across all outcomes, 143 participants (36.9%) had missing outcome data at twelve months. Those with missing data at twelve months were similar in baseline characteristics to those without missing data, although those with missing data were more likely to have obesity than those without missing data (75.4% vs. 67.3%) (Table S3, supplement).

At twelve months, four intervention participants had accessed zero sessions, and 31 had accessed less than four. Overall, 46 participants accessed at least four sessions and 103 accessed at least eight; 62 accessed all twelve sessions.

In the standard advice group, 178 participants completed four-month assessments and were subsequently provided access to the SWiM-C web platform. Of these, most (78/178, 43.8%) did not access any SWiM sessions, and 45 (25.3%) accessed only the first session. Overall, 146 (82%) accessed less than four sessions. Nine participants (5.1%) completed all 12 sessions.

### Primary outcome

**Fig. 2:**
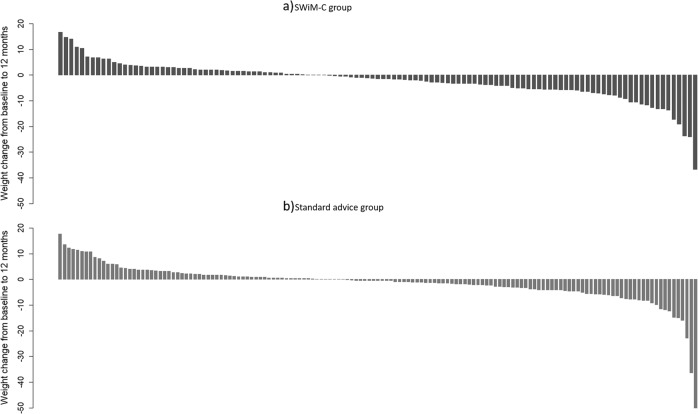
Waterfall graph of weight change. Waterfall graphs showing the distribution of weight change in the (**a**) SWIM-C intervention group (*n* = 119) and the (**b**) standard advice group (*n* = 147).

Participants in the intervention group lost 2.08 kg (SD = 7.30), and participants in the standard advice group lost 1.36 kg (SD = 7.51) from baseline to twelve months (Table 1). The distribution of weight change in the two groups is illustrated in Fig. 2. Following adjustment for baseline weight, sex, and BMI category at baseline, the difference between the groups in weight change from baseline to twelve months was −0.81 kg (95% CI: −2.24 to 0.61). The confidence interval included zero and the results are, therefore, compatible with there being no effect of the intervention on weight change. In the per-protocol analysis, the adjusted difference was −0.30 kg (95% CI: −1.80 to 1.21) between standard advice participants who accessed <4 SWiM sessions and intervention participants who accessed ≥4 SWiM sessions, and −0.70 kg (95% CI: −2.42 to 1.01) between standard advice participants who accessed <4 SWiM sessions and intervention participants who accessed ≥8 SWiM sessions.

**Table 1:** Change in outcomes from baseline to 12 months.

Outcomes		Change from baseline to 12 months (mean, SD)		Baseline-adjusted difference (95% confidence interval)
		N	SWiM-C	
Weight (kg)	SWiM-C	119	−2.08 (7.30)	−0.81 (−2.24 to 0.61)
	SA	147	−1.36 (7.51)	
Depression (Patient Health Questionnaire 8-item scale, PHQ-8, score: 0–24)	SWiM-C	121	−1.36 (4.55)	−0.29 (−1.26 to 0.67)
	SA	152	−1.10 (4.81)	
Anxiety (Generalized Anxiety Disorder 7-item scale, GAD-7, score: 0–21)	SWiM-C	121	−0.17 (3.88)	0.35 (−0.53 to 1.24)
	SA	152	−0.69 (4.56)	
Stress (Perceived Stress Scale 4-items, PSS-4, score: 0–16)	SWiM-C	121	−0.38 (3.22)	0.01 (−0.62 to 0.64)
	SA	152	−0.49 (3.22)	
Experiential avoidance/psychological flexibility (Acceptance and Action Questionnaire Weight Related (Revised), AAQW-R, score: 10–70)^b^	SWiM-C	121	−5.04 (10.99)	**−2.45 (−4.75 to −0.15)**
	SA	151	−2.72 (9.90)	
Eating behaviour (Three-Factor Eating Questionnaire, TFEQ-R21) – Cognitive restraint (score: 0–100)	SwiM-C	121	3.90 (18.24)	1.40 (−1.98 to 4.78)
	SA	152	1.57 (19.38)	
Eating behaviour – Uncontrolled eating (score: 0–100)	SWiM-C	121	−3.20 (14.61)	**−5.52 (−9.67 to −1.37)**
	SA	152	0.99 (15.31)	
Eating behaviour - Emotional eating (score: 0–100)	SWiM-C	121	−7.35 (20.63)	**−4.49 (−7.57 to −1.42)**
	SA	152	−1.64 (19.34)	
Volume of total physical activity (International Physical Activity Questionnaire, IPAQ) in MET-min/week	SWiM-C	101	15.25 (41.40)	**8.96 (0.29 to 17.62)**
	SA	120	6.17 (34.19)	
Wellbeing/capability (ICEpop CAPability measure for Adults, ICECAP-A, score: 0–1)	SWiM-C	121	0.02 (0.12)	0.02 (−0.03 to 0.07)
	SA	151	0.01 (0.15)	

*SA* standard advice, *SD* standard deviation.

Estimates where the confidence interval does not include zero are highlighted in bold.

In the sensitivity analysis assuming MNAR, the difference between groups ranged from −0.66 kg (95% CI: −2.24 to 0.93) to −0.98 kg (95% CI: −3.57 to 1.61) across the seven explored scenarios. Across scenarios, the data were compatible with no effect of the intervention (Table S4, supplement).

Between the four-month and the twelve-month follow-up, SWiM-C participants lost 0.48 kg (SD = 7.47) and standard advice participants lost 0.21 kg (SD = 5.62) (Table 2). The baseline-adjusted difference between groups in change in weight from four months to twelve months was −0.22 kg (95% CI: −1.95 to 1.37).

**Table 2:** Change in outcomes from 4 months to 12 months.

Outcomes		Change from 4 months to 12 months (mean, SD)		Baseline-adjusted difference (95% confidence interval)^a^
		N	SWiM-C	
Weight (kg)	SWiM-C	114	−0.48 (7.47)	−0.22 (−1.95 to 1.37)
	SA	141	−0.21 (5.62)	
Depression (Patient Health Questionnaire 8-item scale, PHQ-8, score: 0–24)	SWiM-C	116	−0.14 (4.24)	0.55 (−0.47 to 1.57)
	SA	148	−0.68 (4.08)	
Anxiety (Generalized Anxiety Disorder 7-item scale, GAD-7, score: 0–21)	SWiM-C	116	0.16 (4.08)	**0.98 (0.005 to 1.95)**
	SA	148	−0.84 (3.83)	
Stress (Perceived Stress Scale 4-items, PSS-4, score: 0–16)	SWiM-C	116	−0.33 (3.02)	0.06 (−0.65 to 0.78)
	SA	148	−0.39 (2.82)	
Experiential avoidance/psychological flexibility (Acceptance and Action Questionnaire Weight Related (Revised), AAQW-R, score: 10–70)	SWiM-C	115	0.93 (8.97)	0.97 (−1.32 to 3.26)
	SA	147	0.01 (9.59)	
Eating behaviour (Three-Factor Eating Questionnaire, TFEQ-R21) - Cognitive restraint (score: 0–100)	SWiM-C	115	−3.48 (19.68)	**−5.38 (−9.61 to −1.16)**
	SA	148	1.05 (19.35)	
Eating behaviour - Uncontrolled eating (score: 0–100)	SWiM-C	115	4.95 (15.15)	0.41 (−3.17 to 4.00)
	SA	148	4.15 (16.05)	
Eating behaviour - Emotional eating (score: 0–100)	SWiM-C	115	−2.76 (18.86)	−3.77 (−8.33 to 0.80)
	SA	148	1.35 (20.91)	
Volume of total physical activity (International Physical Activity Questionnaire, IPAQ) in MET-min/week	SWiM-C	102	14.29 (44.51)	4.52 (−6.69 to 15.72)
	SA	116	9.97 (35.81)	
Wellbeing/capability (ICEpop CAPability measure for Adults, ICECAP-A, score: 0–1)	SWiM-C	121	0.02 (0.12)	0.02 (−0.01 to 0.05)
	SA	151	0.01 (0.15)	

*SA* standard advice.

Estimates where the confidence interval does not include zero are highlighted in bold.

### Secondary outcomes

**Fig. 3:**
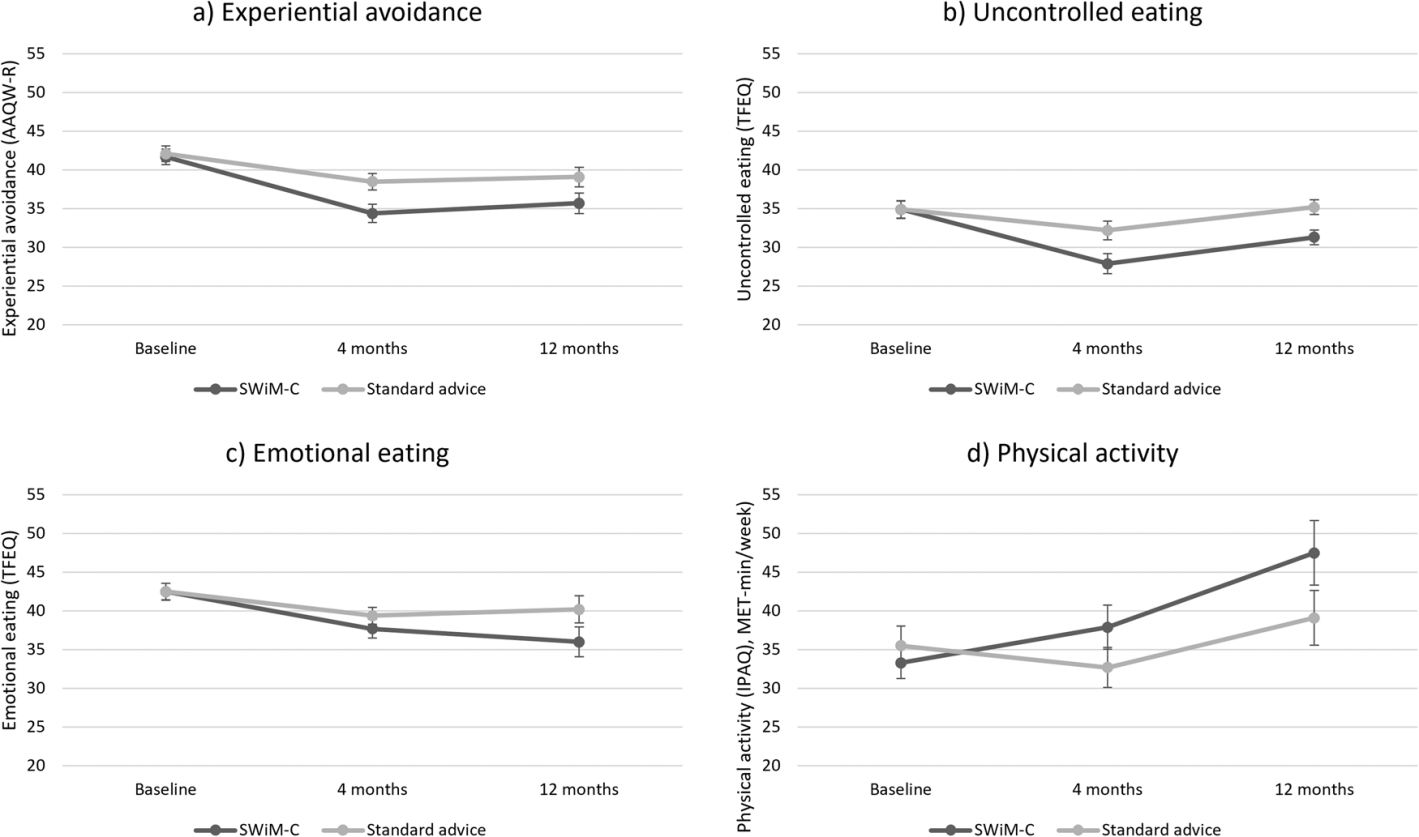
Line graphs for secondary outcomes. Mean experiential avoidance (**a**), uncontrolled eating (**b**), emotional eating (**c**), and physical activity (**d**) in the two study groups at baseline, 4 months and 12 months. Error bars are standard errors. AAQW-R Acceptance and Action Questionnaire Weight Related (Revised), score: 10–70; TFEQ Three-Factor Eating Questionnaire, all subscales score: 0–100; IPAQ International Physical Activity Questionnaire.

From baseline to twelve months, SWiM-C participants reported a greater reduction in experiential avoidance (−2.45, 95% CI: −4.75 to −0.15), uncontrolled eating (–5.52, 95% CI: –9.67 to –1.37), and emotional eating (–4.49, 95% CI: –7.57 to –1.42) as well as a greater increase in physical activity (8.96, 95% CI: 0.29 to 17.62) than standard advice participants (Table 1 and Fig. 3).

From four months to twelve months, there was a small increase in anxiety (0.98, 95% CI: 0.005 to 1.95) and a decrease in cognitive restraint (−5.38, 95% CI: −9.61 to −1.16) in SWiM-C participants compared to standard advice (Table 2; Figure S1 in supplement).

## Discussion

This study evaluated the effect of SWiM-C, an ACT-based, web-based, guided self-help intervention, on weight change over twelve months during the COVID-19 pandemic. We found that SWiM-C improved eating behaviour (reduced uncontrolled and emotional eating), decreased experiential avoidance, and increased physical activity, compared to standard advice.

The difference in weight change between SWiM-C participants and standard advice participants was −0.81 kg following adjustment for covariates. As the confidence interval included zero, it is possible that this difference is due to random chance rather than a systematic effect of the intervention. At four months, the estimated difference between the intervention and the standard advice group was −0.6 kg [15] (though, again, the data were compatible with no effect of the intervention on weight). Therefore, we cannot conclude that the intervention had an effect on weight.

Further refinement and evaluation of the intervention is required before we can consider implementation in practice. For example, increased coach support may improve engagement with and effectiveness of the intervention [30]. We are currently developing and pilot-testing a version of the SWiM intervention that is not specific to the COVID-19 pandemic and that can be implemented in a wide variety of contexts [19, 31].

At twelve months, the effect on uncontrolled eating observed at four months was maintained, with a reduction of –4.82 (95% CI: –7.92 to –1.72) at four months [15] and –5.52 (95% CI: –9.67 to –1.37) at twelve months. This suggests that participants in the intervention group were less likely to lose control over food intake (e.g., feeling unable to stop once one has started eating, or always feeling hungry enough to eat at any time), and this was maintained long-term.

At twelve months, intervention participants reported a lower propensity to overeat in relation to negative mood states than participants who received standard advice [21]. We had observed a small difference in emotional eating at four months [15], though we could not conclude an effect of the intervention at that timepoint. At twelve months, however, this difference increased further and indicated a systematic effect of the intervention on emotional eating.

Similarly, at four months SWiM-C participants reported slightly more physical activity than standard advice participants, but results were inconclusive. At twelve months this difference increased allowing the conclusion that SWiM-C increased physical activity.

One possible explanation for these ‘delayed effects’ may be that ACT requires individuals to learn new skills and techniques, such as ‘cognitive defusion’, that require familiarisation, repeated practice and reflection. They may, therefore, need more than four months to exert full effects. This hypothesis is supported by findings from a systematic review and network meta-analysis which indicated that ACT-based interventions achieved greater weight loss than no/minimal intervention at 9 months, but not at 3 and 6 months [2]. Future research should explore this hypothesis further to elucidate the length of time needed for ACT-based weight-management interventions to exert full effects.

The effect on cognitive restraint we observed at four months [15] was attenuated at twelve months (Fig. S1, supplement). This suggests that, although SWiM-C participants increased their tendency to consciously restrict food intake to control body weight at four months, at twelve months they no longer consciously restricted their food intake. This may reflect a change in participants’ goals from weight loss to maintenance. Alternatively, it is possible that conscious restriction is less necessary when uncontrolled and emotional eating are reduced. We cannot confirm these hypotheses based on the present data, but future research may wish to explore how changes in eating behaviour sub-domains affect one another. Previous research suggests that long-term conscious restriction of energy intake can be extremely challenging, particularly in ‘obesogenic’ environments [32]. Our findings may, therefore, also suggest that participants require more support to maintain cognitive restraint long-term.

The effect of SWiM-C on experiential avoidance that we found at four months was maintained at twelve months, with a reduction of –3.39 (95% CI –5.55 to –1.23) at four months [15] and a reduction of −2.45 (95% CI −4.75 to −0.15) at twelve months. A decrease in experiential avoidance indicates that participants increased their willingness to experience uncomfortable internal experiences (such as food cravings) in order to act in accordance with personal values (e.g., being healthy) [33]. This may in turn have reduced emotional and uncontrolled eating [34, 35].

The fact that effects on uncontrolled eating and experiential avoidance were sustained at twelve months suggests that the SWiM-C intervention had lasting effects on multiple known psychological determinants of weight management. It should be noted that we are not aware of any published definitions of clinically important change for experiential avoidance or eating behaviour subscales. It is therefore difficult to assess whether the changes found in our study are clinically meaningful.

A key strength of the present study is the inclusion of non-weight outcomes. It is important to assess how weight management interventions affect outcomes such as wellbeing and physical activity because these outcomes are associated with important health benefits irrespective of weight loss. For example, wellbeing may have a protective role in health maintenance [36], and physical activity has beneficial physiologic effects independent of weight loss [37].

Standard advice participants in this study were given access to the web-based components of the intervention after completing four-month follow-up assessments to mitigate ethical issues of denying control participants access to treatment. This may have influenced results at the twelve-month follow-up. However, only 18% accessed more than the first four sessions (with ACT-based content introduced from session 4). Accordingly, the per-protocol analysis, which excluded standard advice participants who had engaged with the intervention after being given access, yielded comparable results to the primary analysis. Further limitations of the study include the use of self-reported weight (which may limit accuracy [38]) and the use of social media accounts of obesity and weight management organisations for recruitment (which may limit generalisability), discussed in detail elsewhere [15]. We did not assess if participants were engaged in any other weight management programmes or treatments (e.g. pharmacotherapy) during the study phase and were therefore unable to control for this in our analyses. However, we would expect this to be evenly distributed across the randomised groups. There was a relatively high proportion of missing data at follow-up, with more missing data in the intervention than in the standard advice group. Differential dropout can reduce the validity of findings if completers differ from those who drop out [39]. Findings should, therefore, be interpreted with caution. In the sensitivity analysis assuming data were missing not at random, the difference varied in magnitude but not direction, supporting our overall conclusions.

Finally, generalisability may be limited. Most participants were female, White, and university-educated. Additionally, on average participants reported higher depressive symptom and anxiety symptom scores than found in previous studies with adults with overweight/obesity [40, 41], possibly due to worsening mental health outcomes during the COVID-19 pandemic [16]. While this may limit generalisability, SWiM-C was designed to be used during times of high emotional distress.

## Conclusions

The SWiM-C intervention led to improvements in known determinants of successful weight management over twelve months, including improvements in experiential avoidance, emotional eating, uncontrolled eating, and physical activity. Compared to the four-month follow-up, effects at twelve months were maintained for experiential avoidance and uncontrolled eating, and effects increased for emotional eating and physical activity. These findings suggest that ACT-based interventions can produce longer-term changes and may continue to exert effects on outcomes several months post-intervention. We found no evidence that the intervention reduced weight by 1 kg compared to the control group, but we could not rule out presence of a smaller effect. Findings are promising, but further intervention development and evaluation in large trials is required to assess the potential of remote, web-based interventions drawing on ACT to support weight management.

## Supplementary information

SUPPLEMENTAL MATERIAL

## Data Availability

The dataset analysed during the current study is not publicly available. Participant consent allows for data to be shared in future analyses with appropriate ethical approval, and the host institution has an access policy (https://www.mrc-epid.cam.ac.uk/wp-content/uploads/2019/02/Data-Access-Sharing-Policy-v1-0_FINAL.pdf) so that interested parties can obtain the data for replication or other research purposes that are ethically approved. Data access is available upon reasonable request (datasharing@mrc-epid.cam.ac.uk). Study documents (study protocol, statistical analysis plan, informed consent form, participant information sheet, analytic code) are available at https://www.isrctn.com/ISRCTN12107048 or on request.
