# Correction: Supporting Weight Management during COVID-19 (SWiM-C): twelve-month follow-up of a randomised controlled trial of a web-based, ACT-based, guided self-help intervention

**DOI:** 10.1038/s41366-023-01330-4

**Published:** 2023-07-21

**Authors:** Julia Mueller, Rebecca Richards, Rebecca A. Jones, Fiona Whittle, Jennifer Woolston, Marie Stubbings, Stephen J. Sharp, Simon J. Griffin, Jennifer Bostock, Carly A. Hughes, Andrew J. Hill, Clare E. Boothby, Amy L. Ahern

**Affiliations:** 1 MRC Epidemiology Unit, University of Cambridge, Cambridge, UK; 2 Primary Care Unit, Department of Public Health and Primary Care, University of Cambridge, Cambridge, UK; 3 Patient and Public Involvement Representative, Cambridge, UK; 4 Fakenham Medical Practice, Fakenham, UK; 5 Medical School, University of East Anglia, Norwich, UK; 6 Division of Psychological and Social Medicine, School of Medicine, University of Leeds, Leeds, UK

**Keywords:** Weight management, Lifestyle modification

Correction to: *International Journal of Obesity* 10.1038/s41366-022-01232-x, published online 11 November 2022

The authors have discovered an error in the way the data for three of the secondary outcomes were processed.

The error pertains to the three subscales of the Three Factor Eating Questionnaire. The TFEQ-R21 consists of 21 items, and responses to each of the items were given a score between 1 and 4 and item scores were summated into scale scores for cognitive restraint, uncontrolled eating, and emotional eating. The raw scale scores were then transformed to a 0–100 scale using the formula: ((raw score − lowest possible raw score)/possible raw score range) * 100 (see: de Lauzon et al. The Three-Factor Eating Questionnaire-R18 is able to distinguish among different eating patterns in a general population. J Nutr. 2004;134:2372–80. 10.1093/jn/134.9.2372. PMID: 15333731).

The possible raw score range is the range of the scale (1–4, so 3) multiplied by the number of items. For example, if a participant completed the 6 items on the Restraint Subscale, the possible raw score range is 3 * 6 = 18. Unfortunately, the authors used 4 instead of 3 for the range of the scale, which means that instead of transforming the scores to a 0–100 scale, they transformed the scores to a 0–75 scale. This has now been corrected in the tables below.

This does not affect the overall results and the conclusions remain the same, but, using the corrected scoring method, the authors obtained slightly different numbers for three of their secondary outcomes. The original article has been corrected.

