# Supplementary material for: Supporting Weight Management during COVID-19 (SWiM-C): twelve-month follow-up of a randomised controlled trial of a web-based, ACT-based, guided self-help intervention
Source: Int J Obes (Lond). 2022 Nov 11;47(1):51–9. doi: 10.1038/s41366-022-01232-x (PMC9651901; doi:10.1038/s41366-022-01232-x)
Supplement: Supplementary file 1 — SUPPLEMENTAL MATERIAL [file 41366_2022_1232_MOESM1_ESM.docx]

Supplementary materials

Supporting Weight Management during COVID-19 (SWiM-C): Twelve-month follow-up of a randomised controlled trial of a web-based, ACT-based, guided self-help intervention

Julia Mueller^1^, Rebecca Richards^1^, Rebecca A. Jones^1^, Fiona Whittle^1^, Jennifer Woolston^1^, Marie Stubbings^1^, Stephen J. Sharp^1^, Simon J. Griffin^1,2^, Jennifer Bostock^3^, Carly A. Hughes^4,5^, Andrew J. Hill^6^, Clare Boothby^1^, Amy L. Ahern^1^

^1^ MRC Epidemiology Unit, University of Cambridge, Cambridge, UK

^2^ Primary Care Unit, Department of Public Health and Primary Care, University of Cambridge, Cambridge, UK

^3^ Patient and Public Involvement representative, Cambridge, UK

^4^ Fakenham Medical Practice, Fakenham, UK

^5^ Medical School, University of East Anglia, Norwich, UK

^6^ Division of Psychological and Social Medicine, School of Medicine, University of Leeds, Leeds, UK

*Table S1. Cronbach’s alpha values for the questionnaires used in the study at baseline, 4 months and 12 months.*

| **Questionnaire** | **Baseline** | **4 months** | **12 months** |
| --- | --- | --- | --- |
| Depression (Patient Health Questionnaire 8-item scale, PHQ-8, score: 0-24) | 0.879 | 0.877 | 0.885 |
| Anxiety (Generalized Anxiety Disorder 7-item scale, GAD-7, score: 0-21) | 0.905 | 0.91 | 0.911 |
| Stress (Perceived Stress Scale 4-items, PSS-4, score: 0-16) | -0.312 | 0.085 | -0.253 |
| Experiential avoidance/psychological flexibility (Acceptance and Action Questionnaire Weight Related (Revised), AAQW-R, score: 10-70) ^b^ | 0.89 | 0.896 | 0.919 |
| Eating behaviour (Three-Factor Eating Questionnaire, TFEQ-R21) - Cognitive restraint (score: 0-100) | 0.707 | 0.763 | 0.216 |
| Eating behaviour - Uncontrolled eating (score: 0-100) | 0.849 | 0.871 | 0.612 |
| Eating behaviour - Emotional eating (score: 0-100) | 0.666 | 0.628 | 0.937 |
| Volume of total physical activity (International Physical Activity Questionnaire, IPAQ) in MET-min/week | 0.154 | 0.559 | 0.443 |
| IPAQ – without item on sitting | 0.383 | 0.549 | 0.543 |
| Wellbeing/capability (ICEpop CAPability measure for Adults, ICECAP-A, score: 0-1) | 0.797 | 0.969 | 0.838 |

Table S2. Means and standard deviations (SD) of outcomes at baseline, 4 months, and 12 months by study group. Note: Tables 1 and 2 in the manuscript report change in weight between two timepoints and therefore include only participants who reported a weight value at both timepoints, whereas baseline means in Table S2 include all participants who reported a weight value at each timepoint (hence mean change here differs from the difference between the means reported in Table 2).

| **Outcomes** | | **SWiM-C**  Mean (SD) | | | | | | **Standard advice**  Mean (SD) | | | | | |
| --- | --- | --- | --- | --- | --- | --- | --- | --- | --- | --- | --- | --- | --- |
|  | | **N** | **Baseline^1^** | **N** | **4-months** | **N** | **12-months** | **N** | **Baseline^2^** | **N** | **4-months** | **N** | **12-months** |
| Weight (kg) | | 192 | 98.5 (21.6) | 145 | 96.3 (22.1) | 119 | 95.7 (22.4) | 196 | 98.1 (25.1) | 177 | 95.5 (22.3) | 147 | 96.4 (25.1) |
| Depression (PHQ-8) | | 192 | 7.6 (5.4) | 145 | 6.1 (5.1) | 121 | 6.0 (5.3) | 196 | 7.8 (5.7) | 180 | 7.0 (5.4) | 152 | 6.8 (5.5) |
| Anxiety (GAD-7) | | 192 | 5.3 (4.9) | 145 | 4.5 (4.3) | 121 | 4.9 (5.0) | 196 | 5. 7 (5.1) | 180 | 5.6 (5.2) | 152 | 5.2 (4.9) |
| Perceived stress (PSS-4) | | 192 | 6.5 (2.9) | 145 | 6.3 (2.6) | 121 | 6.0 (3.3) | 196 | 6.6 (3.3) | 180 | 6.3 (3.2) | 152 | 6.3 (3.4) |
| Psychological flexibility/ experiential avoidance | | 192 | 41.7 (14.0) | 143 | 34.4 (14.0) | 121 | 35.7 (14.6) | 196 | 42.1 (14.0) | 178 | 38.5 (14.1) | 151 | 39.1 (15.5) |
| Eating behaviour | Cognitive restraint | 192 | 43.52 (18.62) | 143 | 51.52 (17.88) | 121 | 46.88 (13.11) | 196 | 43.82 (19.65) | 178 | 45.16 (20.56) | 152 | 46.34 (12.56) |
|  | Uncontrolled eating | 192 | 46.56 (21.31) | 143 | 37.18 (20.91) | 121 | 41.73 (13.88) | 196 | 46.58 (20.56) | 178 | 42.92 (21.22) | 152 | 46.97 (15.37) |
|  | Emotional eating | 192 | 56.68 (20.22) | 143 | 50.27 (19.47) | 121 | 48.02 (28.32) | 196 | 56.63 (20.03) | 178 | 52.56 (19.33) | 152 | 53.55 (28.96) |
| Volume of total physical activity | | 171 | 33.3 (26.7)  n = 171 | 130 | 37.9 (32.5) | 113 | 47.5 (44.3) | 166 | 35.5 (32.7)  n = 166 | 149 | 32.7 (31.6) | 136 | 39.1 (41.1) |
| Wellbeing/capability | | 192 | 0.80 (0.16) | 143 | 0.82 (0.17) | 121 | 0.81 (0.17) | 196 | 0.79 (0.18) | 178 | 0.68 (0.33) | 151 | 0.79 (0.18) |


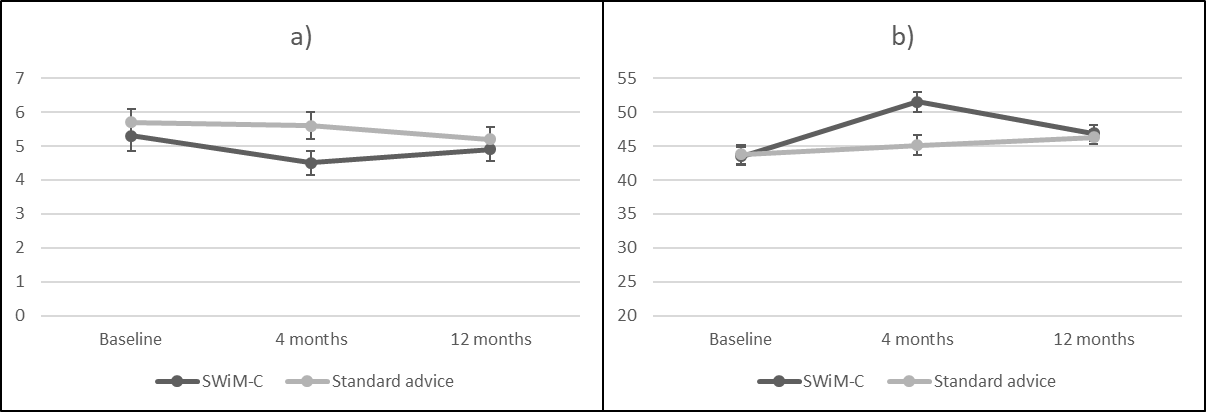


Figure S1. (a) Mean anxiety symptoms as measured using the Generalised Anxiety Disorder questionnaire (GAD-7) and (b) mean cognitive restraint of food intake as measured by the Three Factor Eating Behaviour Questionnaire (TFEQ) at baseline, 4 months and 12 months in the intervention (SWiM-C) group and the standard advice group. Error bars are standard errors.

Table S3. Comparison of participants with and without missing outcome data at 12-month follow-up on demographic characteristics.

|  | **No missing outcome data at 12-month follow-up**,  N = 245  Mean (SD); n (%) | **Missing outcome data at 12-month follow-up**,  N = 143  Mean (SD); n (%) |
| --- | --- | --- |
| Age (years) | 51.44 (13.78) | 48.36 (13.55) |
| Sex (male) | 56 (22.9%) | 28 (19.6%) |
| Ethnicity (White) | 228 (93.1%) | 136 (95.1%) |
| Education (any post-secondary education including university or higher degree or equivalent) | 182 (74.3%) | 11 (69.9%) |
| Marital status (single) | 41 (16.7%) | 26 (18.2%) |
| Weight (kg) | 96.63 (23.15) | 101.12 (23.72) |
| BMI | 34.37 (7.6) | 35.64 (7.93) |
| BMI category (BMI ≥30kg/m^2^) | 165 (67.3%) | 107 (75.4%) |

Table S4. Sensitivity analysis assuming data were missing not at random (MNAR) using pattern mixture models. Missing weight data were imputed using multiple imputation by chained equations (MICE) and were then multiplied imputations by a varying factor (increasing imputations by 0% [MAR], 10%, 20%, 30%, [MNAR] or decreasing imputations by 10%, 20%, 30% [MNAR])).

| **Scenario** | **Baseline-adjusted difference between SWiM-C and standard advice in change in weight from baseline to 12 months (kg)**  **M (95 % CI)** | **p** |
| --- | --- | --- |
| Missing values are 30% smaller than imputed values (MNAR) | -0.66 (-2.24 to 0.93) | 0.40 |
| Missing values are 20% smaller than imputed values (MNAR) | -0.71 (-2.44 to 1.02) | 0.40 |
| Missing values are 10% smaller than imputed values (MNAR) | -0.76 (-2.65 to 1.13) | 0.40 |
| Values are imputed (MAR) | -0.82 (-2.87 to 1.24) | 0.40 |
| Missing values are 10% larger than imputed values (MNAR) | -0.87 (-3.10 to 1.36) | 0.41 |
| Missing values are 20% larger than imputed values (MNAR) | -0.93 (-3.33 to 1.48) | 0.41 |
| Missing values are 30% larger than imputed values (MNAR) | -0.98 (-3.57 to 1.61) | 0.41 |
